# Successful Expulsion of a Golf Ball from the Sigmoid Colon Using Volume Laxatives

**DOI:** 10.1155/2023/5841246

**Published:** 2023-01-06

**Authors:** James P. Grantham, Amanda Hii, Tim Bright, David Liu

**Affiliations:** ^1^Department of Surgery, Royal Adelaide Hospital, Adelaide, SA 5000, Australia; ^2^Oesophagogastric Surgery Unit, Flinders Medical Centre, Bedford Park, SA 5042, Australia; ^3^Division of Surgery, Anaesthesia and Procedural Medicine, Austin Hospital, Heidelberg, VIC 3084, Australia; ^4^Department of Surgery, The University of Melbourne, Austin Precinct, Heidelberg, VIC 3084, Australia; ^5^Department of Surgery, General and Gastrointestinal Surgery Research Group, The University of Melbourne, Heidelberg, VIC 3084, Australia

## Abstract

**Background:**

Rectal foreign bodies form a surprisingly frequent cause of presentation to the emergency department. The materials inserted constitute a wide range of size, shape, and texture with each presenting a unique set of challenges. Despite a seemingly innocuous presentation, if not recognised early and managed accordingly, significant complications can develop including obstruction, perforation, and sphincteric injury. The existing doctrines advocate endoscopic intervention after simple measures fail and advise against the use of laxative therapy due to concerns for complications that may arise. The authors of this study challenge this notion, provided certain conditions are met. *Case Presentation*. We report the case of a 14-year-old boy who inserted a golf ball into his rectum, which subsequently migrated proximally into the sigmoid colon on plain radiographic films. The patient was asymptomatic on presentation, and there was no clinical evidence of bowel injury or mechanical bowel obstruction. Endoscopic removal of the golf ball was pursued under general anaesthesia. Despite protracted efforts, the golf ball was not able to be retrieved endoscopically. In an attempt to avoid aggressive surgery, volume laxatives were administered with successful passage of the golf ball several hours later.

**Conclusions:**

This case discusses the unique technical challenges, which may be encountered when attempting to retrieve a large, spherical, and non-confirming foreign body entrapped above the rectosigmoid junction and how these factors can complicate endoscopic retrieval. The authors advocate that in the absence of a mechanical bowel obstruction, patients with foreign bodies possessing physical properties that are amenable to spontaneous passage, a trial of strong aperients, should be considered first line. The author's contention is that direct escalation to removal of foreign body in theatre can be resource draining and may expose the patient to additional risk.

## 1. Background

A frequent and embarrassing cause of presentation to the emergency department is the patient with a rectal foreign body. It is a phenomenon most commonly observed in younger men [1]. Underlying aetiologies include sexual gratification, smuggling, self-medicating, and accidental trauma [2]. The types of objects vary and may present a difficult conundrum for the treating clinician [3].

In the presence of a rectal foreign body, patients often present with anxiety accompanied by rectal discomfort and fullness. Other symptoms may include abdominal pain, rectal bleeding, or an inability to pass stool or flatus [4]. Clinical workup is aimed at confirming the presence and location of the foreign body, as well as excluding local (perineal and rectal injuries) and regional (colonic obstruction and perforation) complications. These steps include physical examination of the abdomen, perineum, and rectum, and abdominal X-ray supplemented by computed tomography if required, and serum biochemistry including white blood cell count along with a C-reactive protein [5–7].

The patient's anxiety levels, type, and location of the foreign body, as well as the presence of complications, largely dictate the management strategy. Most foreign bodies can be removed trans-anally [8]. In adequately relaxed patients with low-lying objects amenable to simple grasping, retrieval may be achieved within the emergency department using anaesthetic-containing lubrication and straight instruments, such as graspers and forceps [8]. If this is unsuccessful or unsafe, patients can be placed under general anaesthesia with endoscopic removal in theatre [9]. These methods can also be facilitated by laparoscopy [9]. If these strategies are unsuccessful, then intra-abdominal removal of the foreign body may be indicated [4]. Laparotomy can be used in a similar fashion to laparoscopy with milking of the colon and rectum, or a colotomy can be performed for extraction of the foreign body [4]. Intraperitoneal perforation should result in an emergency laparotomy [10, 11]. Depending on the degree of intra-abdominal sepsis, a resection and diverting colostomy may be indicated [12]. Internal examination after a challenging extraction is useful to assess the anal sphincters and rectal mucosa for tearing, bleeding, ischaemia, or perforation [6]. Long-term complications can include faecal incontinence, anorectal fistula formation, and stenosis [12, 13]. Interestingly, the use of laxatives to encourage self-evacuation of the foreign body is not commonly considered. This may be based on a fear of inducing colonic obstruction and perforation [1].

In the following case report, we discuss the strategies employed to retrieve a golf ball entrapped within a patient's sigmoid colon. This scenario resulted in a unique set of technical challenges owing to the physical properties of a golf ball and its anatomical location. These factors rendered traditional retrieval strategies ineffective. This case highlights the role of volume laxatives in the management of non-obstructing spherical foreign objects within the distal colon.

## 2. Case Presentation

**Figure 1:**
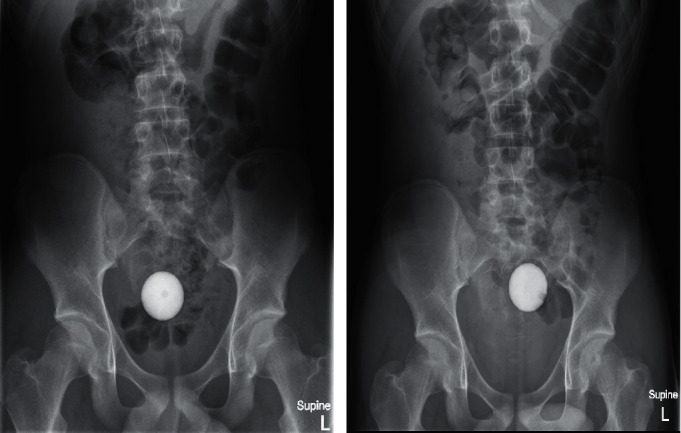
(a) Abdominal X-ray on admission demonstrating the golf ball within the upper rectum. (b) Abdominal X-ray on day 1 inpatient demonstrating proximal migration of the golf ball into the sigmoid colon.

We report the case of a 14-year-old boy with no significant past medical history, who inserted a golf ball into his anus. Upon realising, he was unable to retrieve or pass the object, he notified his mother, and they presented to the emergency department immediately. On presentation, he advised that on self-digitation, he could appreciate the impression of a foreign body within his rectum. He was not in any pain and was able to pass flatus. The patient had attempted to defecate in order to expel the golf ball without success. On examination, there was no evidence of bowel obstruction or trauma to the perineal region. A per rectal examination was not undertaken by the admitting doctor. No abnormalities were detected on full blood examination, biochemistry, liver function tests, or C-reactive protein. A plain abdominal X-ray demonstrated a radiopaque spherical object, consistent with a golf ball, within the patient's pelvis (Figure 1(a)). No cross-sectional imaging, such as computed tomography, was performed due to the radiation exposure risk in the context of the patient's young age.

**Figure 2:**
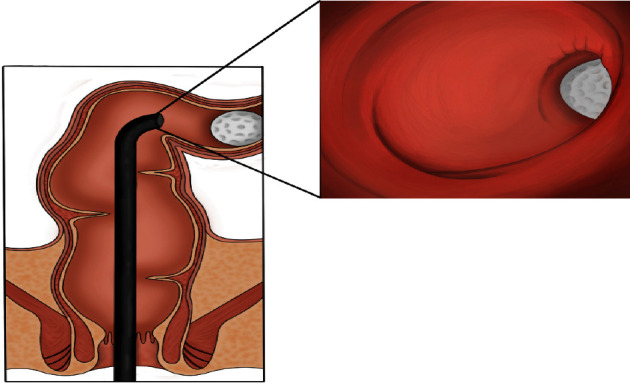
A graphic illustration of the golf ball around the rectosigmoid junction (Tymianski, 2022).

The patient underwent an examination under general anaesthesia, and on rigid sigmoidoscopy, the golf ball was seen located within the sigmoid colon, beyond the reach of straight surgical instruments. We then proceeded to flexible sigmoidoscopy using a range of retrieval devices to remove the golf ball without success. These included a 30 mm Roth Net (Endoscopy, STERIS, USA), a 33 mm single loop CAPTIVATOR II snare (Boston Scientific, Malborough, MA, USA. Steris, Mentor, Ohio Olympus, Shinjuku, Tokyo), a 19.1 mm flexible suction cup (EndoTherapy, Olympus, USA), a quad-prong grasper (Boston Scientific), and a 10 mm Endocatch bag (Autosuture, Medtronic, Minneapolis, MA, USA). We next tried to coax the golf ball back into the rectum by deploying an 18 mm ExtractorPro balloon over a soft-tipped Jagwire (Boston Scientific) just upstream of the golf ball. While this maneuverer was able to mobilise the golf ball to the rectosigmoid junction, the golf ball could not negotiate the acute angle of the rectosigmoid junction and, thus, was not delivered into the rectum. The authors have produced this reconstructive illustration to outline the anatomical challenges encountered in trying to retrieve the golf ball from proximal to the rectosigmoid junction (Figure 2).

Given that our procedure had exceeded 2 hours by this point and that the rectum had been repeatedly instrumented, we decided against further attempts at mechanical retrieval. We speculated that with time, the golf ball may spontaneously enter the rectum. However, an abdominal X-ray performed 24 hours later (Figure 1(b)) demonstrated that the golf ball remained in the sigmoid colon. Taking into consideration the family's reluctance to undergo more aggressive intervention (e.g., laparotomy) and with the absence of colonic obstruction, we decided to trial volume laxatives to facilitate passage of the golf ball. In total, 1 L of glycoprep (Fresenius Kabi, Bad Homburg, Germany) was administered orally, resulting in the successful evacuation of the golf ball 3 hours later. The patient denied experiencing abdominal pain, bloating, or vomiting after consuming the glycoprep. Following passage of the golf ball, the patient remained clinically well and was discharged the same day. There was no evidence of bowel injury. He was advised against inserting further objects into his rectum in the future.

## 3. Discussion

Perhaps the greatest difficulties retrieving rectal foreign bodies stem from the varied nature of materials introduced into a narrow lumen. Its migration into the sigmoid colon presents even greater challenges as the physical distance, and the acute angulation of the rectosigmoid junction limits the effectiveness of straight surgical instruments. Furthermore, endoscopic retrieval devices were not designed to remove large rigid objects. Consequently, each different object offers a unique set of challenges for the endoscopist and, hence, the need for improvisation and ingenuity.

The physical characteristics of a golf ball contribute greatly to the difficulty in attempting trans-rectal retrieval. A standard, commercially available golf ball is spherical, with a diameter of 42.7 mm [14]. On its surface, there are between 250 and 500 circular dimples, which, in normal circumstances, are designed to reduce the drag co-efficient in aerodynamic flight [15]. Most golf balls possess a firm, low friction, thermoplastic resin outer coating [16]. The large size exceeds the breadth of grasping devices, such as the Roth Net, snares, toothed, and hook-prong graspers. Its spherical shape further hinders effort with graspers. The golf ball's firm outer layer renders the object resistant to compression, and thus, achieving optimal conformity with suction devices is difficult. Furthermore, the presence of the dimples on the ball's surface prevents the establishment of an effective seal.

The clinical presentation of a golf ball within the rectum is a phenomenon described only once previously in the medical literature [17]. In this report by Young et al., the object was 15 cm proximal to the anus and was able to be successfully retrieved endoscopically through the use of a 30 mm extraction basket [17]. In our case, the golf ball had migrated proximal to the rectosigmoid junction. The location of a foreign body relative to the rectosigmoid junction has been shown to be a significant determinant in whether or not successful endoscopic retrieval can be achieved [8]. In our experience, this added difficulty stems from the inability of long rigid instruments, such as an Endocatch bag to bend around the rectosigmoid junction, the insufficient length of specific devices to reach more proximally into the colon, and, owing to the acute angulation of the rectosigmoid junction, the entrapment of the golf ball in the sump between the rectal side wall and a more proximally deployed balloon. A double-channel endoscope would have facilitated the use of two instruments simultaneously, and it may be possible that two snares applied to opposite poles of the golf ball would acquire enough purchase on the ball to deliver it into the rectum. However, this would have proven technically challenging, and not all centres have double-channel endoscopes readily available.

Recommendations for removing rectal foreign bodies have generally tended to advise against the use of laxatives due to it being infrequently effective as well as potentially increasing the risk of injury to the bowel [1]. This risk appears to be highly dependent on the mechanical characteristics of the object, with particular caution required for sharp or abrasive objects. It stands to reason that in the setting of an established mechanical bowel obstruction, the administration of laxatives may potentiate further complications. Therefore, in the setting of a smooth and spherical object, located in the colon, that is not causing an obstruction, the authors contend that mobilisation of this object through the action of aperients is unlikely to be deleterious. Moreover, this method of clearance may allow the patient to avoid a general anaesthetic, as well as the risk of iatrogenic injury secondary to prolonged and repeated instrumentation of the rectum [18, 19]. This advice can be extended to any firm and spherical object that may be inserted rectally. In these settings, volume laxatives administered orally may be considered a first-line strategy. A literature review was only able to identify a single publication describing a similar method to facilitate the passage of a rectal foreign body [20]. In this instance, three patients received sodium phosphate or glycerine suppositories to relieve rectally inserted bars of soap. The scarcity of the existing literature detailing this or similar approaches supports the novelty of our recommendations.

In addition to the potentially beneficial outcomes in patient care, there may be healthcare resource advantages to trialling aperients in the first instance. A sachet of standard bowel prep is available commercially in Australia for approximately $10. Conversely, the removal of a rectal foreign body endoscopically can occupy an emergency theatre for a protracted period of time, utilise costly retrieval devices, and draw the endoscopist as well as other staff members away from care to other patients. In situations where the foreign object characteristics are amenable to the administration of laxatives, it seems to be a pragmatic approach to trial their use when theatre availability is limited.

## 4. Conclusions

- A golf ball presents unique technical challenges when attempting to remove from the colon due to its mechanical properties. These include its large size, spherical shape, incompressibility, and the presence of dimples, which prevents a suction seal. The distance and acute angulation of the rectosigmoid junction create additional challenges.
- In the absence of a mechanical bowel obstruction, patients with foreign bodies possessing physical properties that are amenable to spontaneous passage, a trial of strong aperients should be considered first line.
- Direct escalation to removal of foreign body in theatre can be resource draining and may expose the patient to additional risk.

## Data Availability

Data supporting this research article are available from the corresponding author or first author on reasonable request.
